# 

**Published:** 1878-04

**Authors:** 


CONTENTS.

| | Page | | Page |
|---|---|---|---|
| Popular Fallacies | 431 | How to Keep Cool | 448 |
| Architects and Bath Rooms | 432 | Consumption | 448 |
| How to Lend Money | 434 | Summer Complaints | 449 |
| Cause of Death | 435 | Cleanliness | 450 |
| Be Kind to Children | 437 | A Good Wife | 450 |
| Clean Your Cellars | 438 | Summer Traveling | 451 |
| Spirits | 440 | Reward of Labor | 451 |
| Dying Words of Celebrated Persons | 443 | Stimulants | 452 |
| Hints to Pew Owners | 444 | Longevity | 453 |
| The Nature of Cough | 445 | Ventilation | 454 |
| Physical Education of Children | 446 | Wearing the Beard | 456 |
| | | Independent Doctors | 457 |

